# *Plasmodium falciparum* malaria in six travellers returning from Zanzibar to Italy, December 2023 to February 2025: a case series

**DOI:** 10.2807/1560-7917.ES.2025.30.25.2500412

**Published:** 2025-06-26

**Authors:** Claudia Palazzolo, Angela Corpolongo, Serena Vita, Alessandra D’Abramo, Gaetano Maffongelli, Patrizia De Marco, Jingjing Lin, Silvia D’Arezzo, Marco Cilliano, Maria Laura Pettinato, Maria Grazia Bocci, Emanuele Nicastri

**Affiliations:** 1 National Institute for Infectious Diseases “Lazzaro Spallanzani” (IRCCS), Rome, Italy; 2 SS Malattie Infettive, ASL Gallura, Olbia, Italy

**Keywords:** Malaria, Zanzibar, pre-travel consultations, malaria prophylaxis, Italy, Plasmodium falciparum

## Abstract

Between December 2023 and February 2025, six cases of *Plasmodium falciparum* malaria were reported in travellers returning to Italy from Zanzibar, a popular tourist destination often perceived as low risk. None had taken prophylaxis, five had severe malaria and two died. Diagnostic delays revealed a lack of clinical suspicion of malaria and awareness among travellers and healthcare professionals. These cases highlight the importance of malaria chemoprophylaxis and prevention for travellers to risk areas in Africa, clinical suspicion and rapid testing.

Malaria represents an important public health challenge, particularly in low- and middle-income countries. In non-endemic regions, imported malaria poses a diagnostic challenge for healthcare providers, requiring a high index of suspicion when evaluating travellers from endemic areas. Timely diagnosis and rapid treatment are critical to prevent severe complications and death. Severe malaria, if untreated, has a case-fatality rate approaching 100%, while with treatment, it drops to 10–20% [1]. Travelers from non-endemic countries are immunologically naïve and may progress quickly to severe malaria, with high parasite loads and multi-organ involvement [2,3]. Here, we report six cases of *Plasmodium falciparum* malaria in Italy returning from Zanzibar between December 2023 and February 2025. 

## Case series

Of the six cases of *P. falciparum* malaria reported in Italy between December 2023 and February 2025 in returning travellers, five were admitted a specialised infectious disease (ID) clinic in Rome and one to a hospital in Sardinia. The cases were aged between 15 and 55 years, and all patients were of Italian descent without identifiable predisposing risk factors. Four cases had visited Zanzibar for holiday travel and two for work. Diagnosis was confirmed within 15 days of return using rapid diagnostic tests (RDTs) and thick blood smears. In each case, despite recent travel to Zanzibar, malaria was initially not included in the differential diagnosis, leading to a diagnostic delay in five cases of between 2 and 11 days. None of the patients had received malaria chemoprophylaxis; four were discharged in stable condition, while two died within hours of diagnosis. An overview of the six patients is presented in the Table.

**Table:** *Plasmodium falciparum* malaria in travellers returning from Zanzibar to Italy, December 2023–February 2025 (n = 6)

**Case characteristics**	**Case 1**	**Case 2**	**Case 3**	**Case 4**	**Case 5**	**Case 6**
Age in years	40–44	40–44	45–49	20–24	< 20	50–54
Month of travel	Dec 2023	Nov 2024	Oct 2024	Nov 2024	Jan 2025	Feb 2025
Duration of travel (days)	ND	10	11	9	11	8
Malaria prophylaxis	No	No	No	No	No	No
Time from departure to symptom onset	7 days	7 days	8 days	8 days	14 days	2 days
Time from symptom onset to diagnosis	4 days	6 days	0 days	3 days	2 days	11 days
Diagnostic tests	Rapid malaria test	Rapid malaria test	Rapid malaria test	Rapid malaria test	Rapid malaria test	Rapid malaria test
Criteria for severe malaria	Glasgow Coma Scale 3	Alterations in the state of consciousness; coma-shock-MOF-severe metabolic/lactic acidosis	None	Acute respiratory distress syndrome (ARDS)	Severe anaemia	Severe prostration
Laboratory findings at admission	CRP: 11.75 mg/dL; AST/ALT: 92/46 U/L; total/direct bilirubin: 3.76/2.45 mg/dL; blood glucose: 48 mg/dL; creatinine: 1.30 mg/dL; HGB: 10.3 g/dL; D-dimer: ND	CRP: 17.00 mg/dl; AST/ALT: 226/239 U/L; total/direct bilirubin: 19.7/14.3 mg/dL; blood glucose: 101 mg/dL; creatinine: 2.6 mg/dL; HGB: 13.6 g/dL; platelets: 11,000/mmc; D-dimer: 1,266 mcg/dL; pH: 6.96; lactate: 12 mmol/L	CRP: 4.38 mg/dL; AST/ALT: 36/15 U/L; total/direct bilirubin: 0.44/0.14 mg/dL; blood glucose: 101 mg/dL; creatinine: 0.98 mg/dL; HGB: 14.8; D-dimer: 2,216 mcg/dL	CRP: 13.46 mg/dL; total/direct bilirubin: 4.84/2.52 mg/dL; blood glucose: 105 mg/dL; creatinine: 1.12 mg/dL; HGB: 16.7 g/dL; D-dimer: 89,453 mcg/dL	ND	CRP: 24.07 mg/dL; AST/ALT: 97/75 U/L; bilirubin total/direct: 1.22/0.46mg/dL; blood glucose: 128 mg/dL; creatinine: 1.27 mg/dL; HGB: 13.9 g/dL; D-dimer: 149,163 mcg/dL
*Plasmodium*	*P. falciparum*	*P. falciparum*	*P. falciparum*	*P. falciparum*	*P. falciparum*	*P. falciparum*
Parasitaemia	Parasitaemia > 15%	Parasitaemia 12%	Parasitaemia 0.09%	Parasitaemia 0.74%	ND	Parasitaemia 0.08%
Therapy	AS	AS	DHA-PPQ	DHA-PPQ + AS	AS	AS + DHA-PPQ
Outcome from symptom onset	Death at 5 days	Death at 8 days	Discharged at 8 days	Discharged at 19 days	Discharged at 5 days	Discharged at 21 days

AS: artesunate; CRP: C-reactive protein; AST/ALT: aspartate aminotransferase/alanine aminotransferase; DHA-PPQ: dihydroartemisinin-piperaquine; HGB: haemoglobin; MOF: multiple organ failure; ND: not determined.

The data were collected from medical records and reviewed by a trained team of physicians. Norm values for laboratory tests: CRP: 0–5 mg/dL; AST/ALT 5–40 U/L; blood glucose: 70–110 mg/dL; platelets: 150–450,000/mmc; HGB: 12–15.8 g/dL; creatinine: 0.7–1.3 mg/dL; D-dimer < 50 µg/dL; total/direct bilirubin: 0.3–1.2/ 0.1–0.4 mg/dL.

Case 1 was aged between 40–44 years with a history of frequent travel to Tanzania and Zanzibar. They developed fever and altered neurological status 7 days after returning to Italy. Four days after admission, malaria was diagnosed through RDT, thick blood smear and PCR for *P. falciparum*. The patient had parasitaemia over 15% and severe lactic acidosis. Because of worsening neurological status (Glasgow Coma Scale score of 3), they were intubated and transferred to the intensive care unit (ICU). Intravenous artesunate therapy was started, but the patient developed cardiac arrhythmia and died hours later.

Case 2, aged between 40–44 years, developed fever, malaise and asthenia 7 days after a 10-day trip to Zanzibar. The patient had not communicated the trip to the general practitioner, who prescribed antibiotic therapy. Six days later, the patient was admitted to the hospital in a coma with fever and altered consciousness. Blood gas showed severe acidosis (pH 6.96), suggesting multiorgan septic shock. The patient was referred to the ICU of the hospital in Sardinia. Upon learning of their travel history, malaria was suspected by the attending physician. A positive RDT and PCR confirmed *P. falciparum*, with 12% parasitaemia on a blood smear. Despite intravenous artesunate, the patient died 2 days later.

Case 3 was aged between 45–49 years with bronchial asthma who made frequent trips to Tanzania and Zanzibar. They developed fever 7 days after returning. They visited the emergency department (ED) of the ID clinic in Rome, where a positive malaria RDT, blood smear and PCR confirmed *P. falciparum* infection with 0.09% parasitaemia. Treated with oral piperaquine and dihydroartemisinin, the patient had a favourable outcome. The patient was a relative of Case 1.

Case 4, aged between 20–24 years, had travelled to Zanzibar for 10 days. Seven days after returning to Italy, they developed high fever, vomiting and loss of appetite. A malaria RDT was positive, confirmed by blood smear and PCR for *P. falciparum*. The patient was admitted to the ID clinic in Rome and started oral therapy with piperaquine and dihydroartemisinin, but 2 days later, respiratory function worsened, and a chest computed tomography (CT) scan showed ARDS. Treatment was switched to intravenous artesunate, antibiotics, corticosteroids, diuretics and oxygen. The patient’s condition continued to decline, requiring transfer to the ICU for high-flow oxygen therapy. Over several days, their condition improved, and they were discharged after 10 days in good health.

Case 5 was aged less than 20 years who travelled to Zanzibar for 12 days and chose not to take the recommended malaria prophylaxis. Fourteen days after returning, the patient developed fever and fatigue, leading to an ED visit. Malaria was confirmed with a positive RDT, blood smear and PCR for *P. falciparum*. The patient was treated with intravenous artesunate and recovered. One month after discharge, they returned to the ID clinic in Rome for an outpatient follow-up visit, in good clinical condition.

Case 6, aged between 50–54 years and affected by hypertension and celiac disease, travelled to Zanzibar for 8 days. Two days after returning, they developed cough, high fever, chills and intense fatigue, and consulted a general practitioner. After 10 days of persistent fever, weakness, abdominal pain and diarrhoea, the patient sought care at the ED of the ID clinic in Rome. Malaria was confirmed by RDT, blood smear and PCR for *P. falciparum*. The patient was admitted to the ID unit, and because of severe fatigue, was treated with intravenous artesunate; they fully recovered.

## Malaria situation in Zanzibar

The Zanzibar archipelago is a popular tourist destination off the eastern coast of Tanzania with a population of 1.9 million inhabitants. Zanzibar is an endemic area for malaria and was one of the first regions in sub-Saharan Africa to implement malaria control measures, including insecticide-treated nets and indoor spraying. Rapid diagnostic tests (RDTs) and artemisinin-based combination therapy (ACT) have been widely used for case detection and treatment since 2003. Despite these efforts, malaria cases in Zanzibar have rebounded because of changes in malaria epidemiology and to imported infections from mainland Tanzania, where malaria remains highly endemic [4,5].

## Discussion

The reported malaria patients presented here involved individuals who had travelled to Zanzibar for a short stay without taking malaria prophylaxis. The use of chemoprophylaxis in high-risk areas and the awareness of malaria risk in returning travellers are crucial for correct and timely diagnosis and treatment [6]. Zanzibar is located in sub-Saharan Africa, where the risk of contracting malaria is 1,000 times higher than in Asia and South America, chemoprophylaxis is generally recommended by specialists in travel medicine and by national and international health authorities [7,8]. In all cases, travellers should be informed that malaria should always be suspected if fever develops after returning from a tropical country, even if chemoprophylaxis has been correctly followed and proper vector control measures implemented. Moreover, patients should be aware of the importance of reporting recent travel to their doctor, as it can prompt appropriate diagnostic testing [9]. In this context, RDTs are crucial for returning travellers, as they provide timely results that expedite diagnosis and treatment.

Five cases had severe malaria according to the World Health Organization (WHO) criteria, and two died [10]. Cases 1 and 2 had a prolonged interval between symptom onset and diagnosis, at 4 and 6 days, respectively, they had a parasitaemia greater than 5% and both died in the ICU at 5 and 8 days after symptom onset. High parasitaemia is an independent predictor of unfavourable clinical outcome, as shown in a cohort study conducted in France by Bruneel et al [11]. Timely diagnosis and prompt initiation of appropriate therapy prevent the clinical progression to multiorgan failure. The 2023 study by Akafity et al. reviews malaria management in the ICU, stressing early diagnosis, timely antimalarial treatment and complication management. It also underscores the role of diagnostics and supportive care, particularly in resource-limited settings [12]. The remaining four cases were discharged in good condition. The criteria for severe malaria in Case 4 included fatigue and anaemia. Case 6 sought emergency care 11 days after symptom onset despite high fever, intense prostration and a clear epidemiologic link i.e. Zanzibar travel. Case 3, with non-severe malaria, was immediately diagnosed in the travel clinic on the same day that symptoms began. Awareness of malaria risk likely influenced by the recent death of their relative from severe malaria (Case 1) may have contributed to early detection. Nonetheless, the patient had not taken malaria prophylaxis.

Our case series emphasises the importance of a comprehensive medical pre-travel evaluation; a retrospective analysis of EuroTravNet’s longitudinal surveillance data, published in 2015 showed that pre-travel consultation was associated with lower proportionate morbidity from malaria and milder clinical outcomes [13]. Travellers should be educated about malaria chemoprophylaxis and prevention, and made aware that prompt medical attention is needed if symptoms occur during or after travel. As noted by Leder et al., individuals who do not have a pre-travel consultation are at a higher risk of contracting malaria [14]. During the pre-travel visit, travellers can be informed about non-pharmacological preventive measures, such as using mosquito bite protection, including insect repellents, bed nets and protective clothing, in addition to chemoprophylaxis. When followed correctly, antimalarial prophylaxis can reduce the risk of malaria by 90–95% [15]. However, no prophylaxis offers complete protection. Factors such as parasite resistance and the selection of the appropriate medication for the specific destination region are critical in determining the effectiveness of the treatment.

A recent study, conducted in Italy in 2019, reported that over 90% of respondents expressed little or no concern about the risk of contracting infectious diseases while traveling, did not receive any pre-travel advice on preventable illnesses, and identified travel agencies as the primary sources for updating travellers’ knowledge on prophylaxis [16]. Therefore, directly engaging travel agencies and airlines could be an effective strategy to establish a key point of contact with travellers, facilitating better awareness and education on the prevention of infectious diseases in tropical regions, with particular emphasis on malaria risk. Although the incidence of malaria in Zanzibar is relatively low compared to mainland Tanzania, the risk of contracting the disease cannot be overlooked, especially in travellers who do not adopt preventive measures. Promoting adherence to both pharmacological and non-pharmacological malaria prophylaxis is essential by raising travellers' awareness about necessary precautions before travel [17]. However, in Italy, while antimalarial prophylaxis is available in pharmacies with a medical prescription, the cost of the fixed-dose combination of atovaquone and proguanil is not covered by the national health system, resulting in sizeable out-of-pocket expenses for travellers. 

Despite the increase in cases in recent years, the perceived risk of malaria in Zanzibar is often considered low. This misperception may lead to insufficient awareness regarding the need for pre-travel medical consultations and prophylaxis. Furthermore, diagnostic delays are common in non-endemic countries because of the nonspecific nature of symptoms and limited awareness among healthcare providers and patients, which complicates the timely diagnosis and management of imported malaria cases.

## Conclusion

This report underscores the critical importance of ensuring that healthcare professionals possess adequate awareness and training to promptly identify malaria in patients returning from endemic regions. This involves meticulous collection of epidemiological history and proactive assessment of recent travel exposures, which constitute indispensable components for accurate diagnosis and effective management of imported malaria cases.

## Data Availability

All data generated or analysed relating to this study are presented within this published article.
